# Platelet-rich plasma for knee osteoarthritis: a systematic review and meta-analysis of structural outcomes

**DOI:** 10.1007/s00402-026-06543-6

**Published:** 2026-10-22

**Authors:** Eric Pasqualotto, Tales Pasqualotto, Laura Lunelli, Fernando Baldy de Sousa, Luis Fernando Z. Funchal, Fernando Baldy dos Reis

**Affiliations:** 1 Programa de Pós-Graduação em Cirurgia Translacional, Universidade Federal de São Paulo, São Paulo, Brazil; 2 Departamento de Medicina, University Alto Vale do Rio do Peixe, Caçador, Brazil; 3 Departamento de Medicina, Universidade do Sul de Santa Catarina, Palhoça, Brazil; 4 Ortopedia e Traumatologia, Hospital Sancta Maggiore, São Paulo, Brazil; 5 Ortopedia e Traumatologia, Hospital Baía Sul Medical Center, Florianópolis, Brazil; 6 Ortopedia e Traumatologia, Universidade Federal de São Paulo, São Paulo, Brazil

**Keywords:** Platelet-rich plasma, Knee osteoarthritis, Disease-modifying treatment

## Abstract

**Purpose:**

To assess the impact of platelet-rich plasma (PRP) on imaging-based structural outcomes in patients with knee osteoarthritis (OA).

**Methods:**

PubMed, Embase, and the Cochrane Library were systematically searched for randomized controlled trials (RCTs) published up to May 2026 evaluating imaging-based structural effects of PRP in knee OA. Risk ratio (RR) was used to evaluate binary outcomes and standardized mean differences (SMDs) or mean differences (MDs) for continuous endpoints, with 95% confidence intervals (CIs).

**Results:**

A total of 12 RCTs involving 1,200 patients were included. PRP was associated with greater improvement in patellofemoral cartilage thickness/volume compared with control treatments (SMD 0.94; 95% CI, 0.53 to 1.34), whereas no significant differences were observed for medial tibial cartilage thickness/volume or cartilage defect progression. Magnetic resonance imaging (MRI)-based structural scores showed no between-group differences in Whole-Organ Magnetic Resonance Imaging Score (WORMS) osteophytes or subchondral cysts, although PRP demonstrated a significant reduction in bone marrow edema (SMD − 0.40; 95% CI, − 0.71 to − 0.09). Synovitis did not differ overall between groups (SMD − 0.22; 95% CI, − 0.43 to 0.00).

**Conclusion:**

In this systematic review and meta-analysis, PRP demonstrated limited and inconsistent effects on imaging-based structural outcomes in knee OA, and did not confer significant and consistent benefits in medial tibial cartilage measurements, cartilage defect progression, synovitis, or most WORMS domains. The favorable findings observed for patellofemoral cartilage and bone marrow edema should be interpreted with caution. Thus, the findings do not provide clear evidence supporting PRP as a structural disease-modifying treatment, and additional high-quality RCTs using standardized imaging endpoints are warranted.

**Supplementary Information:**

The online version contains supplementary material available at https://doi.org/10.1007/s00402-026-06543-6.

## Introduction

 Knee osteoarthritis (OA) is a highly prevalent degenerative joint disease affecting more than 250 million individuals worldwide, characterized by the progressive deterioration of articular cartilage, subchondral bone, menisci, and periarticular soft tissues [1, 2]. Clinically, knee OA manifests with pain, stiffness, and functional limitation that substantially impair mobility and quality of life [3, 4]. Multiple risk factors, including age, obesity, prior joint injury, malalignment, mechanical overload, and genetic predisposition influence its development and progression [5]. Due to its chronic and debilitating nature, knee OA remains a significant public health problem worldwide.

A broad range of conservative therapies is used to manage knee OA, including nonsteroidal anti-inflammatory drugs (NSAIDs), physiotherapy, weight management, intra-articular corticosteroids, and hyaluronic acid (HA) injections, which typically provide some symptom relief without a disease-modifying effect [6–9]. Platelet-rich plasma (PRP), an autologous concentrate rich in growth factors and cytokines, has received increasing attention for its potential to modulate inflammation, promote cartilage homeostasis, and alleviate symptoms [4, 10]. Despite the growing clinical use of PRP, major guidelines, including those from the American College of Rheumatology (ACR) and the Osteoarthritis Research Society International (OARSI), recommend against its routine use due to weak evidence of outcomes, heterogeneous preparation methods, and methodological limitations in prior trials [7, 8]. Although several systematic reviews report favorable effects of PRP on pain and function compared with saline, corticosteroid, or HA, the quality of evidence remains variable [11–14]. 

Thus, it is still unclear whether PRP confers measurable benefits on structural outcomes, including cartilage thickness, bone marrow lesions, or disease progression. Previous trials have primarily focused on symptomatic endpoints, with structural parameters inconsistently reported [4, 10, 15, 16]. Moreover, head-to-head comparisons with other intra-articular therapies such as HA or corticosteroids have yielded conflicting findings, leaving uncertainty regarding PRP’s potential to alter joint degeneration. Therefore, a comprehensive synthesis of imaging-based structural outcomes is needed. This systematic review and meta-analysis aimed to evaluate the structural effects of intra-articular PRP in patients with knee OA compared with other treatments.

## Methods

This systematic review followed the Preferred Reporting Items for Systematic Reviews and Meta-Analysis (PRISMA) guidelines [17]. The study protocol was registered in the International Prospective Register of Systematic Reviews (PROSPERO) with registration number CRD420251243109.

### Search strategy and data extraction

PubMed, Embase, and Cochrane Library were systematically searched from inception to May 2026, with the following search strategy: (“Platelet-Rich Plasma” OR “PRP” OR “Platelet Rich Plasma”) AND (“Knee Osteoarthritis” OR “Knee OA” OR “Osteoarthritis, Knee” OR KOA OR “Osteoarthritis of the knee”) AND (MRI OR “MR imaging” OR “magnetic resonance” OR Ultrasonography OR Ultrasound*) AND (RCT OR random OR randomly OR randomized OR randomised OR randomization OR “Randomized Controlled Trial” OR “Randomized Clinical Trial”). The search strategies were adapted to the controlled vocabulary of each database. No formal gray literature database search was performed. Conference abstracts were excluded according to the prespecified eligibility criteria. Aiming the inclusion of additional studies, references of the included articles and systematic reviews of the literature were evaluated [18]. Duplicate records were removed during the screening process, and potentially overlapping reports were assessed based on study characteristics. Two authors (E.P. and T.P.) independently extracted baseline characteristics and data outcomes following predefined search criteria: (1) follow-up; (2) nº of patients; (3) age; (4) sex; (5) body mass index; and (6) Kellgren-Lawrence grade. Three authors resolved disagreements by consensus (E.P., T.P., and L.F.Z.F.).

### Eligibility criteria

Studies were included if they met the following criteria: (1) randomized controlled trials (RCTs); (2) comparing intra-articular PRP with other treatments; (3) enrolling patients with clinically or radiographically confirmed knee OA; and (4) reporting at least one structural imaging outcome of interest. We excluded: (1) non-RCTs; (2) studies without structural imaging outcomes; (3) overlapping populations; (4) protocols without published results; (5) conference abstracts; or (6) studies without intervention groups of interest. No restrictions were applied according to PRP preparation subtype.

### Endpoints and subgroup analysis

The outcomes of interest were: (1) medial tibial cartilage change; (2) patellofemoral cartilage change; (3) Whole-Organ Magnetic Resonance Imaging Score (WORMS) score of osteophyte; (4) WORMS score subchondral cyst; (5) synovitis; (6) cartilage defects progression; (7) bone marrow edema; (8) areas of cartilage thinning; (9) improvement at the MRI; and (10) total WORMS score.

### Risk of bias and quality assessment

The Cochrane Collaboration tool for assessing risk of bias in randomized trials (Rob-2) was used to assess the quality of individual RCTs [19]. Each trial received a classification of low risk of bias, some concerns, or high risk of bias in five domains: randomization process; deviations from the intended interventions; missing outcome data; measurement of the outcome; and selection of the reported result. Missing outcome data were assessed within the domain addressing missing outcome data. Two independent authors conducted the risk of bias assessment (E.P. and T.P.) and disagreements were resolved unanimously with the senior author.

Quality of evidence was assessed according to the Grading of Recommendation, Assessment, Development and Evaluation (GRADE) guidelines [20, 21]. Very low, low, moderate, or high-quality evidence grades were designed for the outcomes based on the risk of bias, inconsistency of results, imprecision, publication bias, and magnitude of treatment effects.

### Statistical analysis

The treatment effects for continuous outcomes were compared using weighted mean differences (MDs) or standardized mean differences (SMDs) and binary endpoints were evaluated using risk ratios (RRs), with 95% confidence intervals (CIs). Heterogeneity was assessed with the Cochrane Q-test and I^2^ statistics; P value < 0.10 was considered indicative of statistically significant heterogeneity and I² values were interpreted according to established guidelines [22]. DerSimonian and Laird random-effects models were used for all endpoints [23]. Leave-one-out procedures were used to identify influential studies and their effect on the pooled estimates for outcomes with three or more studies. The Cochrane Handbook for Systematic Reviews of Interventions was used for data handling and conversion [24]. Statistical analyses were performed using R statistical software, version 4.5.2 (R Foundation for Statistical Computing).

## Results

### Study selection and characteristics

**Fig. 1:**
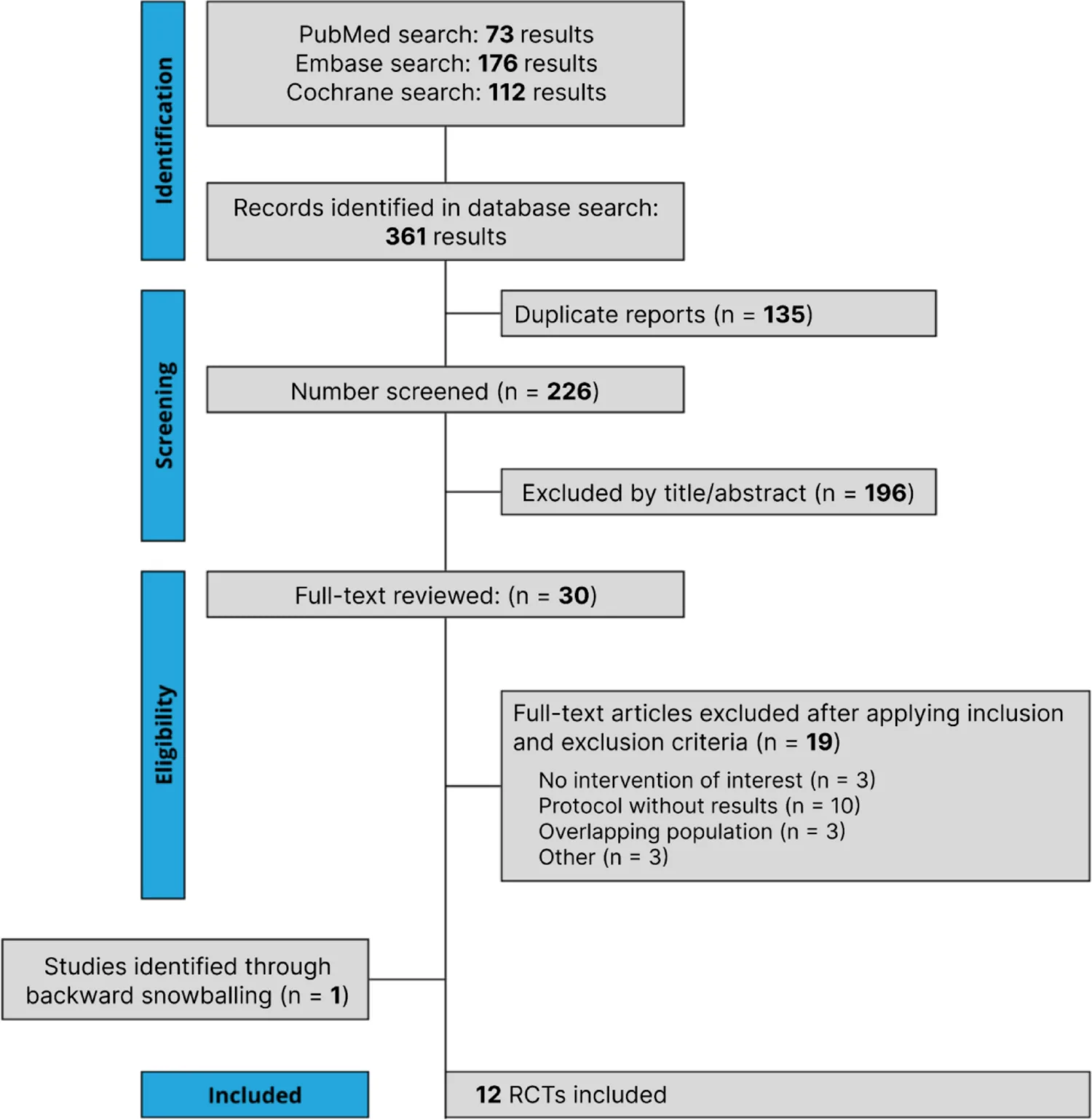
Preferred reporting items for systematic review and meta-analysis (PRISMA) flow diagram of study screening and selection

The initial search yielded 361 results, as detailed in Fig. 1. After removing duplicate records and assessing the studies based on title and abstract, 28 full-text remained and were thoroughly reviewed according to inclusion and exclusion criteria. Of these, 12 RCTs comprising 1,200 patients with knee OA were included [4, 10, 15, 16, 25–32]. 572 (47.7%) patients were randomized to PRP. The mean age was 59.4 years. Study and participant characteristics are summarized in Table 1 and Supplementary Material S1.

**Table 1:** Design and characterístics of studies included in the meta-analysis

Study	Follow-up	Comparison	*N*° of patients, *n* PRP/CG	Age (years), mean ± SD PRP/CG	Female %, PRP/CG	BMI (kg/m²), mean ± SD, PRP/CG	Kellgren Lawrence grade, %, PRP/CG
Ghadamzadeh 2025	6 months	PRP vs. NS	42/21	61.16 ± 6.56✝	NA	28.3 ± 3.07	NA
Raeissadat 2020	8 months	PRP + exercise vs. exercise	23/23*	57.57 ± 5.9✝	100/ 100	28.49 ± 3.24	1: 26.3/26.3 2: 52.6/52.6 3: 21.1/21.1
Bennell 2021	12 months	PRP vs. NS	144/144	62.2 ± 6.3/ 61.6 ± 6.6	59.0/ 58.3	29.0 ± 3.7/ 29.6 ± 4.5	2: 47.9/50.0 3: 52.1/50.0
Buendía-López 2018	52 weeks	PRP vs. HA or NSAID	33/65	56.15 ± 3.0/ 57.03 ± 3.0	51.5/ 52.3	24.9 ± 0.32/ 25.0 ± 0.45	1: 54.5/53.8 2: 45.5/46.2
Zhang 2025	12 months	PRP + HA vs. HA	29/29	57.7 ± 8.0/ 56.7 ± 8.1	44.8/ 44.8	27.0 ± 6.1/ 26.9 ± 3.1	NA
Lisi 2017	6 months	PRP vs. HA	30/28	53.5 ± 15.1/ 57.1 ± 10.0	33.0/ 43.0	NA	NA
Lin 2023	3 months	PRP vs. SH	45/36	52.8 ± 3.2/ 54.2 ± 4.1	71.1/ 75.0	26.2 ± 0.71/ 25.4 ± 0.65	2: 71.1/69.4 3: 28.9/30.6
Zaffagnini 2022	24 months	PRP vs. MF-AT	55/53	54.1 ± 10.6/ 54.5 ± 12.1	34.5/ 71.4	28.0 ± 5.5/ 25.9 ± 4.3	1: 16.4/15.1 2: 29.1/37.7 3: 32.7/24.5 4: 21.8/22.6
Tschopp 2024	NA	PRP vs. HA, NS or glucocorticoid	32/92	60.70 ± 10.50✝	NA	NA	NA
Ahmad 2018	6 months	PRP vs. HA	45/44	56.2 ± 6.8/ 56.8 ± 7.4	68.9/ 68.2	26.7 ± 3.6/ 26.5 ± 3.5	1: 17.8/ 15.9 2: 37.8/ 43.2 3: 44.4/ 40.9
Bansal 2021	12 months	PRP vs. HA	75/75	64.2 ± 4.59/ 65.5 ± 3.96	39/ 38.2	NA	1: 3.1/ 4.4 2: 12.5/ 13.2 3: 84.3/ 82.3 4: 0/0
Elik 2019	6 months	PRP vs. NS	30/30	61.30 ± 7.91/ 60.19 ± 6.80	96.7/ 88.9	30.37 ± 4.47/ 30.70 ± 3.97	1: 6.6/11.1 2: 46.7/48.1 3: 16.7/40.7

*Number of knees. ✝The mean refers to the total number of patients included in the study

*SH* sodium hyaluronate, *NS* normal saline, *PRP* platelet-rich plasma, *HA* hyaluronic acid, *MF-AT* microfragmented adipose tissue, *CG* control group, *NA* not available, *SD* standard deviation, *NSAID* nonsteroidal anti-inflammatory drugs

### Pooled analysis of all studies

#### Cartilage changes

**Fig. 2:**
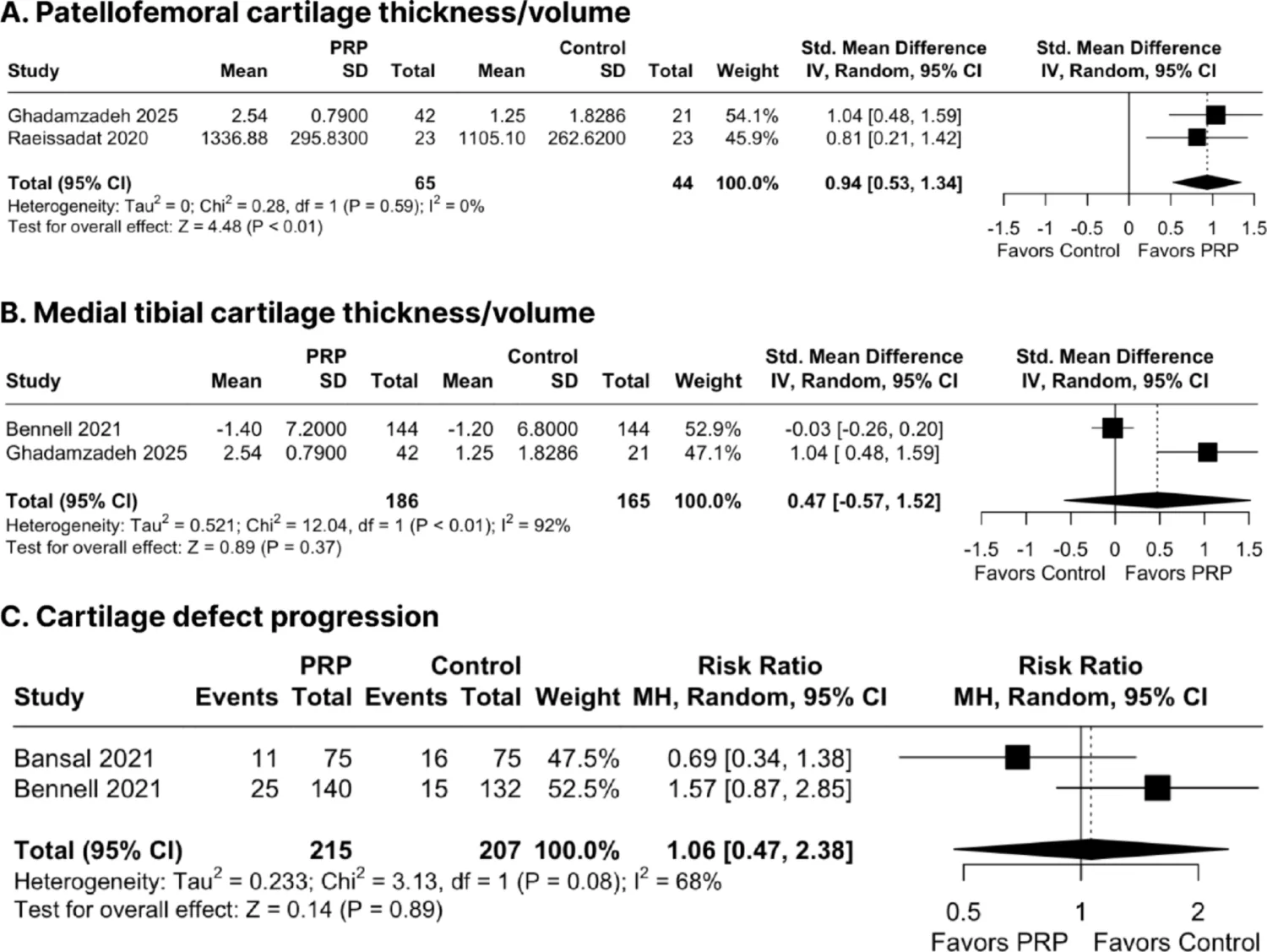
Forest plots of pooled comparisons between platelet-rich plasma (PRP) versus other treatments for knee osteoarthritis. **A** Patellofemoral cartilage thickness/volume. **B** Medial tibial cartilage thickness/volume. **C** Cartilage defect progression

In our pooled analysis, there was a significant difference in favor of the PRP group compared with other treatments in patellofemoral cartilage thickness/volume (SMD 0.94; 95% CI 0.53 to 1.34; *p* < 0.01; I^2^ = 0%; Fig. 2A). However, no significant difference was observed between groups for medial tibial cartilage thickness/volume (SMD 0.47; 95% CI −0.57 to 1.52; *p* = 0.37; I^2^ = 92%; Fig. 2B). Similarly, the aggregated data showed no significant between-group differences in cartilage defect progression (RR 1.06; 95% CI 0.47 to 2.38; *p* = 0.89; I^2^ = 68%; Fig. 2C).

While Lisi (2017) reported ≥ 1-grade MRI improvement in 48.3% of patients treated with PRP compared with only 8% of controls (*P* < 0.003), Bennell (2021) found a higher proportion of PRP-treated participants with ≥ 3 areas of cartilage thinning relative to placebo (17.1% vs. 6.8%; RR 2.71; 95% CI, 1.16–6.34) [10, 31]. Morphological parameters showed no significant changes between groups at 3 and 12 months in Tschopp (2024) [28]. 

#### WORMS and MRI structural scores

**Fig. 3:**
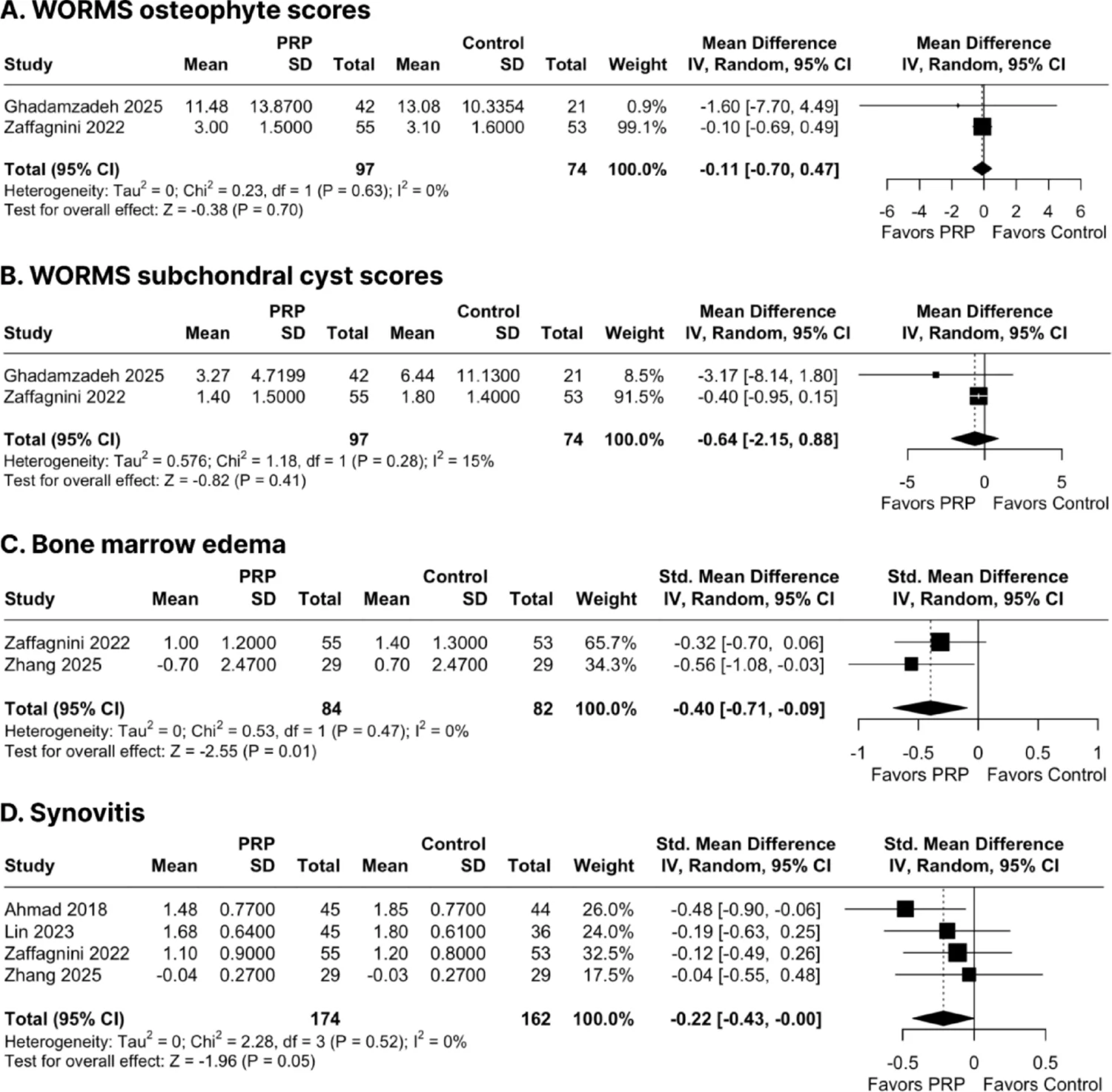
Forest plots of pooled comparisons between platelet-rich plasma (PRP) versus other treatments for knee osteoarthritis. **A** WORMS osteophyte scores. **B** WORMS subchondral cyst scores. **C** Bone marrow edema. **D** Synovitis

In our pooled analysis, no significant between-group differences were observed in WORMS osteophyte scores (MD −0.11; 95% CI −0.70 to 0.47; *p* = 0.70; I^2^ = 0%; Fig. 3A) or WORMS subchondral cyst scores (MD −0.64; 95% CI −2.15 to 0.88; *p* = 0.41; I^2^ = 15%; Fig. 3B). However, there was a significant difference in bone marrow edema in favor of the PRP group (SMD − 0.40; 95% CI −0.71 to −0.09; *p* = 0.01; I^2^ = 0%; Fig. 3C).

Consistent with these findings, Zhang (2025) reported similar reductions in total WORMS scores at 12 months in both the HA + PRP and HA-alone groups, with no meaningful difference between treatments [4]. 

#### Synovitis and inflammatory findings

There was no significant difference between groups in synovitis when assessed using synovitis scoring systems (SMD − 0.22; 95% CI −0.43 to 0.00; *p* = 0.05; I^2^ = 0%; Fig. 3D).

Individual trials, however, reported heterogeneous findings. Raeissadat (2020) observed a greater reduction in synovitis in the PRP group compared with the exercise control group (28.5% vs. 52.3%) [29]. Bansal (2021) found low and comparable rates of persistent synovitis between PRP and HA-treated patients (4.6% vs. 10.2%) [30]. 

### Sensitivity analysis

We performed a leave-one-out sensitivity analysis for the synovitis outcome, which demonstrated that omitting Zhang (2025) results in a significant reduction in synovitis in favor of the PRP group (Supplementary Material, Figure S1) [4]. 

### Risk of bias

**Fig. 4:**
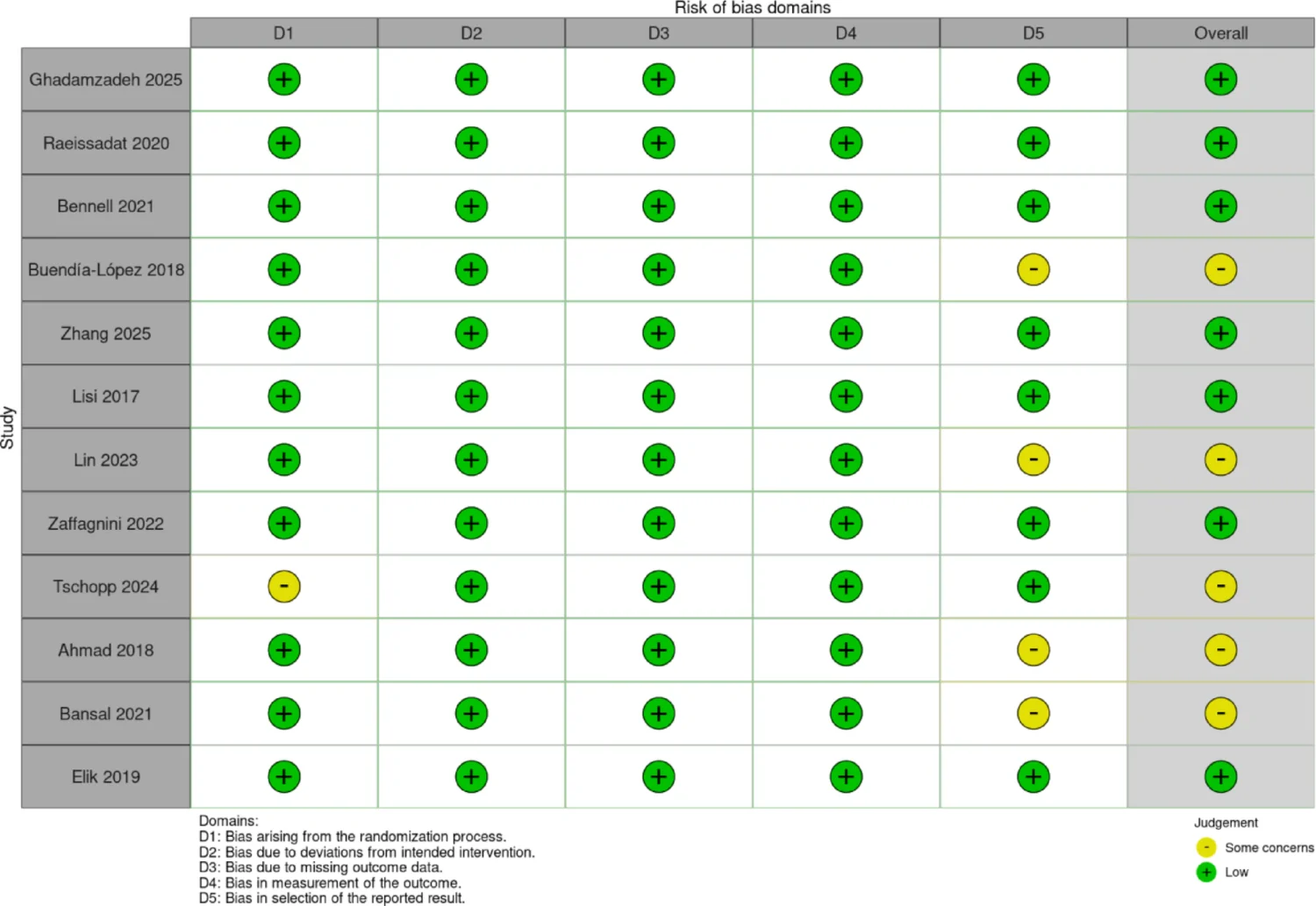
Critical appraisal of randomized controlled trials according to the cochrane collaboration’s tool for assessing risk of bias in randomized trials

Figure 4 presents the detailed risk-of-bias assessment for each RCT included in the meta-analysis. Overall, five studies were rated as having some concerns for risk of bias, whereas seven studies were judged to have a low risk of bias. The domain with the greatest number of concerns was bias in selection of the reported results, reflecting potential issues related to selective reporting or insufficiently pre-specified outcomes.

 According to the GRADE assessment, five outcomes evaluated in this study were classified as low-quality evidence: patellofemoral cartilage thickness/volume, WORMS osteophyte scores, WORMS subchondral cyst scores, bone marrow edema, and synovitis. Two outcomes had very low quality of evidence: medial tibial cartilage thickness/volume and cartilage defect progression. The main reasons responsible for reducing the quality of evidence across the outcomes were the use of different measures to evaluate the endpoints, variation in control groups, the small number of studies reporting each outcome, high heterogeneity, and the presence of some concerns for risk of bias. Quality assessment is detailed in the Supplementary Material, Table S2.

## Discussion

In this systematic review and meta-analysis of 12 RCTs involving 1,200 patients with knee OA, intra-articular PRP demonstrated limited and inconsistent effects on structural joint outcomes compared with other treatments. Although PRP was associated with greater improvement in patellofemoral cartilage thickness/volume and a reduction in bone marrow edema, no significant benefits were observed in medial tibial cartilage measures, cartilage defect progression, or WORMS-derived osteophyte and subchondral cyst scores. For synovitis, the pooled analysis did not show a robust between-group difference, and this result was sensitive to excluding a single study in the leave-one-out analysis, indicating uncertainty rather than a confirmed absence of effect. However, the certainty of evidence was low to very low for all structural outcomes assessed, limiting confidence in the pooled estimates and precluding conclusions regarding structural disease modification.

Current evidence suggests that PRP may offer clinically meaningful improvements in pain and function for patients with knee OA, particularly when compared with HA, corticosteroid, or placebo [12, 13]. Prior meta-analyses have reported reductions in pain and enhanced functional scores at short- and mid-term follow-up [33]. However, these symptomatic improvements have not been accompanied by high-certainty evidence of structural modification [34]. Trials that incorporated MRI- or ultrasound-based endpoints have reported heterogeneous and generally neutral findings, with most failing to demonstrate measurable preservation or improvement of cartilage morphology [4, 10, 16, 29, 30]. Notably, large, well-conducted trials such as RESTORE observed clinical improvement with PRP but found no significant differences in medial tibial cartilage volume compared with placebo, a result consistent with our pooled estimates [10]. 

This study represents, to our knowledge, the first meta-analysis to quantitatively pool structural imaging outcomes of PRP for knee OA based exclusively on RCTs. The findings of this meta-analysis align with key studies that similarly reported neutral or modest structural effects of PRP. Zaffagnini et al. (2022) likewise observed no changes across multiple WORMS components, including cartilage morphology, BME, osteophytes, and meniscal integrity, up to 24 months [27]. Buendía-López et al. (2018) reported comparable radiographic progression across PRP and HA groups, suggesting that PRP does not substantially alter structural trajectories detected by conventional imaging [15]. These studies collectively support the conclusion that PRP performs similarly to other treatments in terms of disease modification.

Other studies, however, have suggested potential localized or modest structural improvements following PRP. Lisi et al. (2017) demonstrated ≥ 1-grade MRI improvement in nearly half of PRP-treated patients compared with 8% of controls, and our pooled analysis showed a significant benefit in the patellofemoral compartment; however, the certainty of evidence for this outcome was classified as low by GRADE, which limits confidence in the magnitude and generalizability of this finding [31]. Bone marrow edema, a known correlate of pain and disease activity, improved significantly in two RCTs of Zhang et al. (2025) and Lin et al. (2023) and contributed to a favorable pooled effect [4, 26]. This finding, however, warrants careful interpretation, as bone marrow edema and bone marrow lesions represent a biological construct distinct from cartilage preservation. These subchondral and inflammatory imaging features are potentially reversible and considerably more dynamic over time than cartilage morphology, and their improvement should therefore be regarded as a marker of reduced inflammatory or subchondral activity rather than as evidence of cartilage preservation or of attenuated structural progression of OA. Additionally, Tschopp (2024) reported regional improvements in T2 mapping values in select compartments when comparing PRP with corticosteroids and placebo [28]. These isolated signals suggest that PRP may exert biological activity in specific tissue domains or subregions. Nevertheless, the inconsistent magnitude and anatomical specificity of these effects, along with the predominance of neutral findings in larger RCTs, indicate that these benefits remain insufficient to establish PRP as a structural disease-modifying intervention.

The clinical implications of these findings warrant careful consideration. Although PRP is widely used and continues to attract interest as an orthobiological intervention, the available evidence does not support its use as a structural disease-modifying therapy for knee OA, especially due to very low- to low-quality evidence provided by our meta-analysis. Furthermore, the present findings highlight the need for rigorously designed trials employing standardized imaging protocols, uniform PRP preparation methods, and harmonized definitions of structural endpoints to more definitively determine whether PRP exerts any disease-modifying effects.

This study has several limitations. First, although only RCTs were included, many had small sample sizes, contributing to inaccuracy and limiting the power to detect subtle structural changes. Second, heterogeneity was substantial for some outcomes, especially medial tibial cartilage measures, reflecting differences in comparators, imaging techniques, PRP preparation protocols, and follow-up durations among the included studies; with the available data, it was not possible to isolate a predominant source. Accordingly, the pooled estimate for this outcome should be interpreted with considerable caution, rather than as a single, consistent effect of PRP. Furthermore, MRI-based structural assessment was not standardized across trials, with differences in acquisition sequences, scoring systems, anatomical regions assessed, and quantitative versus semiquantitative measurements, which may have contributed to between-study heterogeneity. Third, structural outcomes were inconsistently reported, reducing the number of studies eligible for each pooled analysis. Cartilage thickness and cartilage volume were combined using SMD, which allows statistical integration of measures on different scales but does not guarantee full biological equivalence between the two parameters; this combination constitutes an additional methodological limitation, and the principal finding for the patellofemoral compartment should be interpreted in light of this caveat. Fourth, the comparator groups included in the pooled analyses were clinically and pharmacologically heterogeneous (normal saline, HA, corticosteroid, NSAIDs, exercise, and microfragmented adipose tissue), which precludes interpreting the estimates as reflecting a single, uniform effect of PRP against a common comparator. The limited number of studies per comparator category did not allow for statistically robust subgroup analyses, and the pooled estimates should therefore be interpreted with additional caution. In addition, PRP is not a biologically standardized intervention, and characteristics such as leukocyte content, platelet concentration, activation method, injected volume, and number of injections varied among the included studies, often without complete reporting. This heterogeneity in PRP composition and administration protocol may have contributed to the inconsistency of the structural findings and limits the interpretation of the results as a single, uniform intervention. Finally, follow-up durations were relatively short, ranging between 3 and 24 months, which may be insufficient to detect disease-modifying effects in slowly progressive conditions such as OA.

## Conclusions

In this systematic review and meta-analysis, PRP demonstrated limited and inconsistent effects on imaging-based structural outcomes in knee OA, and did not confer significant and consistent benefits in medial tibial cartilage measurements, cartilage defect progression, synovitis, or most WORMS domains. The favorable findings observed for patellofemoral cartilage and bone marrow edema should be interpreted with caution, given the low certainty of evidence, the limited number of contributing studies, the heterogeneity of comparators, and the relatively short follow-up duration. Thus, the findings do not provide clear evidence supporting PRP as a structural disease-modifying treatment, and additional high-quality RCTs using standardized imaging outcomes are needed to reach a definitive conclusion.

## Supplementary Information

Below is the link to the electronic supplementary material.

Supplementary Material 1

## Data Availability

All data supporting the findings of this study are available within the paper and its Supplementary Information.
