# Supplementary material for: Platelet-rich plasma for knee osteoarthritis: a systematic review and meta-analysis of structural outcomes
Source: Arch Orthop Trauma Surg. 2026 Oct 22;146(1):381. doi: 10.1007/s00402-026-06543-6 (PMC13690889; doi:10.1007/s00402-026-06543-6)
Supplement: Supplementary file 1 [file 402_2026_6543_MOESM1_ESM.docx]

**Supplementary Material**

**Figure S1.** Leave-one-out sensitivity analysis plot of the synovitis outcome.

**Table S1.** Platelet-Rich Plasma Protocols and Imaging Assessment Methods in the Included Studies

| **Study** | **PRP preparation** | **Imaging assessment** |
| --- | --- | --- |
| Ghadamzadeh 2025 | PRP was prepared using the Rooyagen Kit (Arya Mabna Tashkhis Corporation; RN: 312569). For each patient, 35 cc of venous blood was collected and mixed with 5 cc of anticoagulant before being distributed into four tubes. For single-spin preparation, samples were centrifuged at 1600 rpm for 10 minutes, after which the upper half of the plasma was separated and 5 cc from the lower plasma fraction was used for injection. For double-spin preparation, plasma obtained after the first centrifugation was transferred to two additional tubes and centrifuged at 3500 rpm for 6 minutes. Following removal of the upper plasma fraction, 3 cc from the bottom of each tube was used for injection. A 0.5-cc sample was obtained at each preparation stage for laboratory analysis. | MRI using coronal, sagittal, and axial fat-suppressed proton-density, T1-weighted, and T2-weighted sequences. |
| Raeissadat 2020 | PRP was prepared using the Rooyagen Kit (Arya Mabna Tashkis Corporation; RN: 312569). Approximately 35–40 mL of venous blood was collected using an 18-G needle and mixed with 5 mL of ACD-A. One milliliter was used for complete blood count, while the remaining sample underwent two centrifugation steps, first at 1600 rpm for 15 minutes and then at 2800 rpm for 7 minutes. The final product consisted of 4–6 mL of leukocyte-containing PRP with a platelet concentration 4–6 times higher than normal values. PRP quality and quantity were assessed using a Sysmex KX 21 analyzer and visual swirling before injection. | Knee MRI using a 1.5-Tesla Siemens AVANTO system with a circumferential knee coil, including transverse 3D TRUFISP and coronal and sagittal fat-saturated proton-density sequences |
| Bennell 2021 | PRP was prepared at each weekly treatment visit using a commercial Regen Lab SA system, with a single centrifugation performed at 1500g for 5 minutes. | Knee MRI using 3.0-Tesla systems at the Melbourne (Philips Ingenia) and Sydney (Siemens Skyra) sites, including T1-weighted 3D gradient-echo sequences and sagittal, axial, and coronal fat-suppressed proton-density (PD) sequences. |
| Buendía-López 2018 | Each patient received 5 mL of PRP prepared from 60 mL of peripheral blood collected from the antecubital vein. A double-centrifugation protocol was used, with an initial spin at 1050 rpm for 15 minutes followed by a second spin at 2000 rpm for 10 minutes. This yielded 5 mL of leukocyte-poor PRP, which was activated with 1 mL of calcium chloride. Platelet concentration was assessed in five randomly selected patients and averaged 1,095,000 ± 23,200/mm³, corresponding to 3.87 times the baseline concentration. | Knee MRI using a 1.5-Tesla Siemens MAGNETOM Essenza system with an extremity coil, including sagittal proton-density (PD) and T2-weighted spin-echo sequences, and coronal and axial fat-suppressed PD and T2-weighted spin-echo sequences. |
| Zhang 2025 | Autologous HA+PRP was prepared using the Cellular Matrix A-CP HA system (Regen Lab SA, Le Mont-sur-Lausanne, Switzerland). Each tube contained 2 mL of non-crosslinked HA gel (1550 kDa; 20 mg/mL), a polymeric separator gel, and sodium citrate. The closed system allowed collection of 6 mL of blood and automated PRP preparation. Centrifugation at 1500×g separated the blood components, with the separator gel retaining erythrocytes and pro-inflammatory leukocytes while allowing plasma and platelets to remain above the gel. Approximately 3 mL of leukocyte-poor PRP was obtained, with a platelet concentration 1.5–1.6 times the baseline blood value. The PRP was mixed with approximately 2 mL of HA by gentle agitation, yielding approximately 5 mL of the final preparation. | Knee MRI using clinical 1.5-Tesla GE Healthcare or Philips Healthcare scanners with dedicated knee coils, including sagittal, coronal, and axial fat-suppressed 2D fast spin-echo (FSE) proton-density-weighted sequences and a sagittal high-resolution intermediate-weighted 2D FSE sequence. |
| Lisi 2017 | Patients received three intra-articular injections of autologous PRP activated with calcium gluconate at 4-week intervals. At each visit, 20 mL of venous blood was collected and mixed with 2 mL of Anticoagulant Citrate Dextrose Solution A. The sample was centrifuged at 900 rpm for 7 minutes, after which PRP was collected and transported to the rehabilitation unit for injection by an experienced physiatrist. | MRI findings were evaluated according to the modified Shahriaree Classification System. |
| Lin 2023 | Ninety milliliters of peripheral venous blood was collected in tubes containing 3.8% sodium citrate. Samples underwent single-spin centrifugation at 500g for 8 minutes at room temperature, producing erythrocyte, PRP, and platelet-poor plasma layers. The intermediate PRP layer was collected into sterile tubes, yielding approximately 8–10 mL with a platelet concentration 1.5–2 times that of peripheral blood and no leukocytes. Platelets were activated with 10% calcium chloride immediately before injection. All PRP preparations were performed by the same orthopedist. | MRI using coronal spin-echo T2-weighted sequences to evaluate subchondral bone marrow edema (BME) and synovitis. BME was quantitatively assessed at the maximum-area section using ImageJ, and synovitis was graded from 0 to 3 according to the volume of enhancing synovial tissue. |
| Zaffagnini 2022 | A single 150-mL unit of peripheral blood was collected in a citrate-phosphate-dextrose-adenine blood bag. Two centrifugation steps were performed, initially at 1480 rpm for 6 minutes to separate erythrocytes and subsequently at 3400 rpm for 15 minutes to concentrate platelets. Platelet-poor plasma was manually removed after the second spin, yielding 5 mL of PRP targeted to a platelet concentration of 1000 × 10³/µL ± 20%. PRP was stored at −30°C, thawed at 37°C for 30 minutes before use, and transported to the outpatient clinic in a thermal bag. The final product had a platelet concentration approximately 5 times higher than whole blood and a leukocyte concentration approximately 1.5 times higher than baseline. | High-resolution (1.5 T) MRI |
| Tschopp 2024 | PRP was prepared using the ACP System (Arthrex, Munich, Germany), producing leukocyte-poor plasma with a platelet concentration of approximately 500 × 10³/µL. | Knee MRI using a 3-Tesla Siemens Skyra-Fit system with a dedicated knee coil, including transverse proton-density fat-saturated, coronal T1-weighted, sagittal T2-weighted 3D isovoxel, T2 mapping, and T2* mapping sequences |
| Ahmad 2018 | Eight milliliters of peripheral blood was collected and centrifuged at 3500 rpm for 9 minutes using a single-spin protocol. Approximately 4 mL of PRP was obtained from each patient for intra-articular injection. The PRP preparation contained leukocytes. | Power Doppler US |
| Bansal 2021 | Sixty milliliters of blood was collected with 10% ACD and centrifuged at 600×g for 10 minutes. The plasma fraction was then separated and centrifuged at 4000×g for 15 minutes. After removal of platelet-poor plasma, 3 mL of PRP remained. Platelets from the PPP fraction were recovered using a 1-µm filter and combined with the initial PRP. The preparation was subsequently passed through a WBC filter to remove leukocytes. Platelet concentration was adjusted to 10 billion cells in 8 mL, using PPP for dilution when necessary. The PRP was not activated, and platelet and leukocyte concentrations were assessed. Five randomly selected samples were additionally analyzed for PDGF and VEGF using ELISA. | MRI wT2 maps |
| Elik 2019 | PRP was prepared using a two-step centrifugation protocol with tubes A and B. Ten cc of blood was collected into tube A containing sodium citrate. The sample was centrifuged at 2000 rpm for 10 minutes, producing erythrocyte, buffy coat, and plasma layers. Plasma and buffy coat were transferred to tube B and activated with 10% calcium chloride. A second centrifugation was performed at 4000 rpm for 5 minutes, yielding approximately 4 mL of PRP. Platelet concentration was approximately 4–6 times higher than whole blood (900,000–1,100,000/mm³), while the leukocyte concentration was approximately equivalent to that of peripheral blood (5000–10,000/mm³). | Ultrasonography using a linear probe of 7–12 Mhz 228  (Esaote MyLab Five) |

**Table S2.** The Grading of Recommendation, Assessment, Development and Evaluation (GRADE) assessment of each endpoint

| **Certainty assessment** | | | | | | | **Summary of findings** | | | | |
| --- | --- | --- | --- | --- | --- | --- | --- | --- | --- | --- | --- |
| **Participants (studies) Follow-up** | **Risk of bias** | **Inconsistency** | **Indirectness** | **Imprecision** | **Publication bias** | **Overall certainty of evidence** | **Study event rates (%)** | | **Relative effect (95% CI)** | **Anticipated absolute effects** | |
|  |  |  |  |  |  |  | **With Control** | **With PRP** |  | **Risk with Control** | **Risk difference with PRP** |

**Patellofemoral cartilage thickness/volume**

| 109  (2 RCTs) | not serious | not serious | serious^a,b^ | serious^c^ | none | ⨁⨁◯◯  Lowa,b,c | 44 | 65 | - | - | SMD **0.94 SD higher** (0.53 higher to 1.34 higher) |
| --- | --- | --- | --- | --- | --- | --- | --- | --- | --- | --- | --- |

**Medial tibial cartilage thickness/volume**

| 351  (2 RCTs) | not serious | serious^d,e^ | serious^a^ | serious^c^ | none | ⨁◯◯◯  Very lowa,c,d,e | 165 | 186 | - | - | SMD **0.47 SD higher** (0.57 lower to 1.52 higher) |
| --- | --- | --- | --- | --- | --- | --- | --- | --- | --- | --- | --- |

**Cartilage defect progression**

| 422  (2 RCTs) | serious^f^ | serious^e^ | serious^b^ | serious^c^ | none | ⨁◯◯◯  Very lowb,c,e,f | 31/207 (15.0%) | 36/215 (16.7%) | **RR 1.06**  (0.47 to 2.38) | 31/207 (15.0%) | **9 more per 1.000**  (from 79 fewer to |
| --- | --- | --- | --- | --- | --- | --- | --- | --- | --- | --- | --- |
|  |  |  |  |  |  |  |  |  |  |  | 207 more) |

**WORMS osteophyte scores**

| 171  (2 RCTs) | not serious | not serious | serious^b^ | serious^c^ | none | ⨁⨁◯◯  Lowb,c | 74 | 97 | - | 74 | MD **0.11 points lower**  (0.7 lower to 0.47 higher) |
| --- | --- | --- | --- | --- | --- | --- | --- | --- | --- | --- | --- |

**WORMS subchondral cyst scores**

| 171  (2 RCTs) | not serious | not serious | serious^b^ | serious^c^ | none | ⨁⨁◯◯  Lowb,c | 74 | 97 | - | 74 | MD **0.64 points lower**  (2.15 lower to 0.88 higher) |
| --- | --- | --- | --- | --- | --- | --- | --- | --- | --- | --- | --- |

**Bone marrow edema**

| 166  (2 RCTs) | not serious | not serious | serious^b^ | serious^c^ | none | ⨁⨁◯◯  Lowb,c | 82 | 84 | - | - | SMD **0.4 SD lower** (0.71 lower to 0.09 lower) |
| --- | --- | --- | --- | --- | --- | --- | --- | --- | --- | --- | --- |

**Synovitis**

| 336  (4 RCTs) | serious^g^ | not serious | serious^b^ | not serious | none | ⨁⨁◯◯  Lowb,g | 162 | 174 | - | - | SMD **0.22 SD lower**  (0.43 lower to 0 ) |
| --- | --- | --- | --- | --- | --- | --- | --- | --- | --- | --- | --- |

**Explanations: (a)** Different measures were used to assess cartilage improvement. (b) The control groups varied between studies. (c) Only two studies reported this outcome. (d) There is no overlap in the confidence intervals between the studies. (e) High heterogeneity. (f) One RCT was classified as having some concerns for risk of bias. (g) Two RCTs were classified as having some concerns for risk of bias. **CI:** confidence interval; **MD:** mean difference; **RR:** risk ratio; **SMD:** standardised mean difference
